# The interplay between DNA damage response and RNA processing: the unexpected role of splicing factors as gatekeepers of genome stability

**DOI:** 10.3389/fgene.2015.00142

**Published:** 2015-04-15

**Authors:** Chiara Naro, Pamela Bielli, Vittoria Pagliarini, Claudio Sette

**Affiliations:** ^1^Department of Biomedicine and Prevention, University of Rome Tor Vergata, Rome, Italy; ^2^Laboratory of Neuroembryology, Fondazione Santa Lucia, Rome, Italy

**Keywords:** genome stability, DNA damage prevention, DNA repair, RNA metabolism, splicing factors, RNA binding proteins, DNA repair proteins

## Abstract

Genome integrity is constantly threatened by endogenous and exogenous factors. However, its preservation is ensured by a network of pathways that prevent and/or repair the lesion, which constitute the DNA damage response (DDR). Expression of the key proteins involved in the DDR is controlled by numerous post-transcriptional mechanisms, among which pre-mRNA splicing stands out. Intriguingly, several splicing factors (SFs) have been recently shown to play direct functions in DNA damage prevention and repair, which go beyond their expected splicing activity. At the same time, evidence is emerging that DNA repair proteins (DRPs) can actively sustain the DDR by acting as post-transcriptional regulator of gene expression, in addition to their well-known role in the mechanisms of signaling and repair of the lesion. Herein, we will review these non-canonical functions of both SFs and DRPs, which suggest the existence of a tight interplay between splicing regulation and canonical DNA safeguard mechanisms ensuring genome stability.

## Introduction

Genome stability in eukaryotic cells is continuously challenged by several endogenous and exogenous factors threatening the integrity of their DNA. Defects in the replication process and exposure to the oxidant agents produced by cellular metabolism are among the possible endogenous causes of DNA damage (Altieri et al., 2008). Dangers for DNA integrity may also originate from recombination events occurring during developmentally regulated processes, such as meiosis (Cooper et al., 2014) or lymphocyte development (Alt et al., 2013), and from defects in the processing of nascent RNAs (Chan et al., 2014). In particular, altered mRNA processing promotes the formation of R-loops, the three-stranded DNA:RNA hybrids that represent one of the major endogenous sources of genome instability (Chan et al., 2014). Environmental stresses, such as those caused by ultraviolet (UV) or ionizing irradiation (IR) and chemicals, can be accounted among the exogenous causes of DNA damage (Altieri et al., 2008; Aguilera and García-Muse, 2013).

Preserving genome integrity is of vital importance for the health of eukaryotic cells, as it ensures the correct transmission of the genetic information during replications. Indeed, alterations of genome integrity, such as DNA mutations, chromosomal rearrangements or aneuploidy, are among the main causes of human pathologies, including hereditary diseases and spontaneous cancers. For these reasons, eukaryotic cells are endowed with several cellular devices deputed to the prevention of DNA damage, such as anti-oxidant scavengers and enzymatic systems counteracting endogenous free-radicals (Rizzo et al., 2010) or the complex network of structural and functional proteins regulating correct chromosomes segregation during mitosis and meiosis (Duro and Marston, 2015). In addition, efficient sensor systems enable cells to promptly detect any eventual DNA insult and in turn activate proper cellular responses to overcome the genotoxic stress. The elicited DNA damage response (DDR) allows cells to arrest cell cycle progression and to activate the appropriate repair system. If the defect is properly resolved, cells resume their normal cell cycle, otherwise cellular senescence or apoptosis of the damaged cells are induced (Ciccia and Elledge, 2010). The correct functionality of the sensors and effectors of the DDR is therefore essential to ensure genome stability and to avoid detrimental consequences for cellular homeostasis. In line with this notion, several pathologies are correlated with deficiencies in the DDR, including neurological and immunological diseases and cancer (Jackson and Bartek, 2009).

The DDR triggers also a broad rearrangement of the gene expression program of the damaged cells, mainly aimed at sustaining the expression of genes involved in the DNA repair, cell-cycle control and/or apoptosis. Importantly, a large part of this reprogramming is mediated by post-transcriptional mechanisms regulating mRNA processing and metabolism (Boucas et al., 2012). A large body of evidence has indeed shown that the expression of several genes involved in the DDR is controlled by mechanisms regulating their splicing profile, the stability of their transcripts and/or their utilization by the translational machinery (Dutertre et al., 2011; Boucas et al., 2012).

A major role in the DDR-elicited control of gene expression is played by alternative splicing (AS). Genotoxic stresses set in motion an extensive AS regulation affecting both mRNA stability and protein activity of genes having critical functions in the DDR (for a detailed review, see Dutertre et al., 2011). This splicing reprogramming relies on the regulation of the expression and activity of specific RNA binding proteins (RBPs) through different mechanisms that have been extensively described elsewhere (Busà and Sette, 2010; Lenzken et al., 2013).

Unexpectedly, a number of studies have now revealed splicing-independent functions in the DDR for many RBPs, which appear to act as gatekeepers of genome integrity or as proper transducers and effectors of the DDR. On the other hand, recent evidence has highlighted a role in splicing regulation for some of the proteins directly involved in the detection, signaling, and repair of the DNA damage. Herein, we will discuss some of the examples of splicing factors (SFs) and DNA repair proteins (DRPs) having dual role in AS and in maintenance of genome stability and DDR.

## Splicing Factors in the Prevention and Repair of DNA Damage

Genome-wide screenings aimed at the identification of proteins involved in the maintenance of genome stability and proper induction of the DDR have revealed an unexpected enrichment for proteins involved in pre-mRNA processing (Paulsen et al., 2009; Hurov et al., 2010; Adamson et al., 2012). Interestingly, the crucial role of these RBPs in the preservation of genome integrity often goes beyond their functions in post-transcriptional control of gene expression through AS. Several SFs seem to be directly involved in the prevention and repair of DNA damage through multiple mechanisms. Thus, it is presently unclear whether the splicing-dependent or -independent activities of these RBPs, or a combination of both, are involved in the DDR.

A clear example is provided by the Ewing Sarcoma protein (EWS). Two independent high-throughput screenings identified the gene encoding EWS (*EWSR1*) as required for resistance to IR (Hurov et al., 2010) and camptothecin (O’Connell et al., 2010). In line with this observation, *Ewsr1* knockout mice and embryonic fibroblasts were highly susceptible to IR (Li et al., 2007). In addition, investigation of the phenotype of these mice demonstrated an unpredicted role of EWS in homologous recombination (HR) during B cell development and meiosis (Li et al., 2007). The specific role played by EWS in the DDR and HR was not elucidated. However, *in vitro* studies had previously shown that EWS promotes the annealing of homologous DNA (Guipaud et al., 2006), which is an essential step in both HR and DNA double-strand breaks (DSBs) repair, suggesting that EWS might play a direct role in these processes. Nevertheless, EWS also regulates AS of genes involved in the DDR and apoptosis (Dutertre et al., 2010; Paronetto et al., 2011, 2014) and its splicing activity is modulated in response to both irradiation (Paronetto et al., 2011) and camptothecin (Dutertre et al., 2010). Thus, it is also possible that a combination of splicing-dependent and splicing-independent functions of this RBP promote resistance of eukaryotic cells to genotoxic stresses.

**FIGURE 1:**
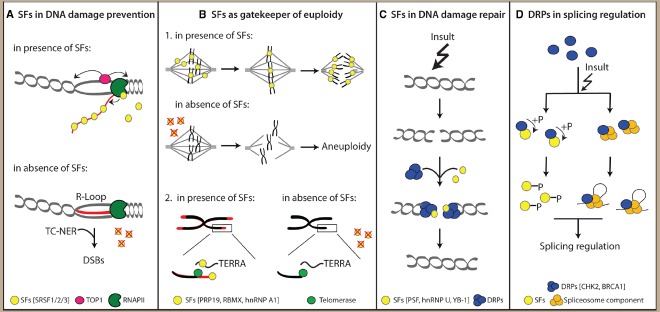
**Non-canonical functions of splicing factors (SFs) and DNA repair proteins (DRPs) ensure genome stability. (A)** SFs prevent transcription-related DNA insults such as R-loop formation. **(B)** SFs act as gatekeeper of cellular euploidy by regulating correct chromosomes segregation and preservation of telomeres integrity. **(C)** SFs actively participate to DNA repair processes. **(D)** DRPs regulate pre-mRNA splicing.

Importantly, pre-mRNA splicing and processing factors have also emerged as a prominent portion of the cellular proteome regulated during the DDR by post-translational modifications, such as phosphorylation (Matsuoka et al., 2007; Smolka et al., 2007; Beli et al., 2012), parylation (Jungmichel et al., 2013), and acetylation (Beli et al., 2012). Collectively, these observations strongly suggest that RBPs are often involved in the maintenance of genome stability and establishment of a correct DDR. Below, we will review some specific examples supporting a role of multiple RBPs in various aspects of the response to genotoxic stresses, which can be basically classified in: prevention of RNA-processing defects leading to genome stability, maintenance of chromosomal integrity and sensing/repairing the DNA lesion (Figure 1).

### Splicing Factors Preventing RNA-Induced Genome Instability

One of the dangerous situations that might threaten genomic integrity is represented by the formation of R-loops. During transcription, the nascent RNA can anneal to the transcribed DNA strand and generate a three-stranded nucleic acid structure, named R-loop (Aguilera and García-Muse, 2012). This structure is constituted of an RNA:DNA hybrid and an exposed single strand DNA (Westover et al., 2004). Although R-loops are generated in some physiological conditions (Yu et al., 2003; Skourti-Stathaki et al., 2011; Ginno et al., 2012), these structures are considered dangerous because they might promote mutations, recombination, and chromosome rearrangements (Aguilera and García-Muse, 2012; Hamperl and Cimprich, 2014; Skourti-Stathaki and Proudfoot, 2014). Notably, eukaryotic cells have developed strategies to prevent R-loops formation during DNA transcription, which partly rely on the activity of specific RBPs.

For instance, the serine-arginine (SR) rich protein SRSF1, the prototype of SR family of splicing regulators (Long and Caceres, 2009), plays a key role in preventing the formation of R-loops (Li and Manley, 2005). SRSF1-depleted cells revealed a hypermutagenic phenotype caused by accumulation of R-loops (Li and Manley, 2005), which can then be converted in DSBs by the transcription-coupled nucleotide excision repair (TC-NER) protein Cockayne syndrome group B (CSB; Sollier et al., 2014). Two other SR-proteins, SRSF2/SC35 and SRSF3/SRp20, were shown to be able to suppress R-loop formation (Li and Manley, 2005), further corroborating the involvement of SR proteins in preservation of genomic integrity. These observations suggest that the recruitment of SR-proteins by the RNA polymerase II (RNAPII) during transcription of nascent pre-mRNAs couples splicing of the introns with prevention of aberrant RNA:DNA hybrid structures and consequent DSBs formation (Figure 1A).

The control of R-loop formation by RBPs might, however, be more complex than initially expected and involve multimolecular complexes formed on the nascent transcript. This complexity was initially suggested by the observations that a lot of binding partners of Topoisomerase 1 (TOP1) were proteins involved in RNA metabolism (Czubaty et al., 2005). The most abundant class of TOP1-interacting proteins was represented by SFs, such as SRSF1, p54nrb, the polypyrimidine tract-binding protein-associated splicing factor (PSF), HuR, and several heterogeneous ribonucleoproteins (hnRNPs A2/B1, A1, A0, U, K, and R; Czubaty et al., 2005). TOP1 promotes single-strand DNA (ssDNA) cleavage and re-ligation during RNA transcription and DNA replication, thus allowing relaxing of positive and negative supercoils (Leppard and Champoux, 2005; Chen et al., 2013). Depletion of TOP1 induces DNA damage and replication defects caused by a co-transcriptional accumulation of R-loops (Tuduri et al., 2009). One possibility is that TOP1 promotes R-loop resolution by recruiting SRSF1, and/or other RBPs, to these sites. Alternatively, since TOP1 possesses kinase activity and specifically phosphorylates SR proteins, including SRSF1 (Rossi et al., 1996; Labourier et al., 1998), it is possible that it acts by enhancing the activity of SR proteins locally at sites of R-loop formation (Figure 1A). This hypothesis is supported by the observation that inhibition of TOP1 kinase activity resembles defects in TOP1- or SRSF1-depleted cells (Tuduri et al., 2009). Since the kinase activity of TOP1 is also required for spliceosome assembly and for proper AS of a number of genes (Pilch et al., 2001), its functional interaction with SR proteins might also efficiently couple resolution of R-loops with correct splicing of the pre-mRNA. The interplay between TOP1 and SR proteins might be even more extensive, as it was shown that interaction of SRSF1 with TOP1 inhibits its DNA cleavage activity (Andersen et al., 2002). This interaction seems to be regulated by poly-ADP ribose (PAR; Malanga et al., 2008). In the absence of PAR, SRSF1 interacts with TOP1 and inhibits its DNA cleavage activity while favoring its kinase activity; however, binding of SRSF1 to PAR releases TOP1 and promotes its DNA cleavage activity, while reducing TOP1-mediated SRSF1 phosphorylation (Malanga et al., 2008).

The functional cooperation between TOP1 and SR proteins in the suppression of R-loop formation during transcription is also suggested by the interaction of this enzyme with B52/SRp55, a *Drosophila melanogaster* SR protein (Juge et al., 2010). Upon heat shock (HS), B52/SRp55 is required for recruitment of TOP1 to actively transcribed HS genes, which is followed by a rapid shut down of transcription after an initial burst (Juge et al., 2010). However, depletion of B52/SRp55, or TOP1, impairs *HSP70* mRNA release from its transcription site and disappearance, suggesting that these two proteins participate to the negative feedback of HS gene transcription (Juge et al., 2010). Although not demonstrated yet, this observation suggest that recruitment of TOP1 to actively transcribed genes by B52/SRp55 might be required to release the transcribed RNA, thus limiting the possibility of R-loop formation.

The importance of proper coordination between DNA- and RNA-processing pathway to prevent genome instability is also supported by recent observations involving RBPs other than SR proteins in such regulation, like the yeast hnRNP named NPL3 (Santos-Pereira et al., 2013). Finally, a comprehensive view of the involvement of RBPs in the maintenance of genome integrity was highlighted by a genome-wide siRNA screening (Paulsen et al., 2009). Using as read-out phosphorylation of H2AX (γ-H2AX), a hallmark of DNA damage (Mah et al., 2010), it was shown that the class of genes whose depletion induced γ-H2AX mainly belonged to mRNA processing factors. Interestingly, this induction was alleviated by over-expression of RNaseH, corroborating the idea of an involvement of RBPs in the suppression of R-loop formation and preservation of genome stability.

In conclusion, it is now clear that SFs play a pivotal function in the maintenance of genome integrity by integrating processes at the crossroad between transcription and pre-mRNA processing.

### Splicing Factor Acting as Chromosomal Integrity Gatekeeper

Telomeres represent an important challenge for the maintenance of chromosomal integrity, due to their progressive erosion at each replication and their possible recognition as DNA breaks by repair systems. Telomere integrity is ensured by telomerase holoenzyme activity, mediating their replication, and by the shelterin complex, which protects them from inappropriate DNA repair (O’Sullivan and Karlseder, 2010). Interestingly, several SFs have been shown to be involved in both protection and replication of telomeres, thus unveiling another possible role for these proteins as gatekeeper of genome integrity (Figure 1B). A prototypical example of SF involved in telomere maintenance is hnRNP A1. The first evidence suggesting a role for hnRNP A1 in the regulation of telomeres replication arose from a study from LaBranche et al. (1998), which highlighted shortened telomeres in mouse cell lines depleted for hnRNP A1. It was then shown that hnRNP A1 is able to bind *in vivo* telomeric DNA and that it can enhance telomerase activity, probably by unwinding G-quadruplex inhibitory structures (Zhang et al., 2006). Moreover, it was also suggested that hnRNP A1 ability to concomitantly interact with telomeric DNA and the telomerase RNA component, hTR, may facilitate telomerase recruitment along telomeric regions (Fiset and Chabot, 2001). More recently, interaction between hnRNP A1 and TERRA, the non-coding RNAs transcribed from telomeric regions, has been suggested to regulate telomere coating by POT1, a protein of the shelterin complex, following replication (Flynn et al., 2011). In addition to hnRNP A1 several other hnRNPs have also been implicated in telomere metabolism, because of their association with the telomerase complex, such as hnRNP C and hnRNP U (Fu and Collins, 2007), or with the TERRA RNAs, such as hnRNP A/B, F (López de Silanes et al., 2010). Furthermore, RBPs not belonging to the hnRNP family have also been involved in telomere maintenance. A nice example is offered by Fused-in-Sarcoma (FUS, also known as TLS—Translocated in liposarcoma), a multifunction RNA/DNA binding protein implicated in multiple steps of both DNA and RNA processing, including AS (Dormann and Haass, 2013). FUS interacts with G-quadruplex formed by telomeric DNA and by TERRA and regulates heterochromatin formation at telomeric regions (Takahama et al., 2013). Lastly, a possible role in telomere metabolism has also been suggested for the accessory SF U2AF65, which was shown to regulate TRF1 protein levels, another shelterin, by inhibiting its ubiquitin-mediated degradation (Kim and Chung, 2014). The abilities of SFs to regulate different aspects of telomere metabolism strongly suggests that exploitation of their versatile multifunctional nature represents an important tool for the preservation of chromosomal integrity.

Another possible risk factor for genome stability is represented by defects in chromosome replication and segregation during cell divisions, which may negatively affect euploidy. One of the first hints suggesting the role of SFs in the regulation of correct chromosome segregation was provided by the results of an interference screening aimed at the identification of genes essential for cell division (Kittler et al., 2004). Unexpectedly, this screening revealed defects in the mitotic spindle assembly following depletion of several SFs, suggesting their possible role as regulator of mitosis (Kittler et al., 2004). This study suggested a more direct role for RBPs in mitosis, in addition to their canonical influence on this process exerted through proper splicing of some key mitotic regulators (Mu et al., 2014; Sundaramoorthy et al., 2014; Watrin et al., 2014). For example, some SFs have now been implicated in the maintenance of spindle integrity, such as hnRNP U (also known as SAF-A; Ma et al., 2011) and the spliceosomal component PRP19 (Hofmann et al., 2013; Figure 1B). Cells depleted for either for hnRNP U or PRP19 showed spindle abnormalities, due to defects in the interaction between kinetochores and microtubules (Ma et al., 2011; Hofmann et al., 2013). Intriguingly, the spindle defects observed following hnRNP U depletion could be caused by an impaired recruitment of Aurora A mitotic kinase at the spindle microtubule (Ma et al., 2011). Furthermore, SFs appear to be involved in the regulation of sister chromatid cohesion. A strict cohesion of sister chromatids along the entire length of duplicated chromosomes is required until metaphase; then, prior to mitosis, cohesion is restricted to the centromeric region in order to ensure proper chromosome alignment and prevent aneuploidy (Peters and Nishiyama, 2012). Notably, the hnRNP protein RBMX (also known as hnRNP G) was reported to promote centromeric localization of SGO1, a key regulator of the centromeric cohesion of sister chromatids (Matsunaga et al., 2012).

In conclusion, these results provide strong evidence for a direct role of SFs as gatekeeper of cellular euploidy, operated through their control of both proper structural organization and accurate segregation of chromosomes.

### Splicing Factors Involved in the DNA Repair

Some SFs were shown to be recruited at DNA damage sites and to reduce sensitivity of cells to genotoxic stresses (Li et al., 2009; Salton et al., 2010; Mastrocola et al., 2013), indicating their direct participation in the DNA repair process.

In several cases, the recruitment of SFs to the DNA lesions appeared to be dependent on PAR polymerase (PARP) activity (Adamson et al., 2012; Mastrocola et al., 2013; Britton et al., 2014; Rulten et al., 2014). PARPs act as molecular sensors for both single- and double-strand DNA breaks and their activity is crucial for signaling the DNA-lesion and for activation of proper DDR. PAR chains added by PARPs on chromatin proteins act as docking sites for the recruitment of DNA repair complexes (Sousa et al., 2012). Notably, a large part of the PAR-interacting proteome is composed by RBPs (Gagné et al., 2012) and a set of SFs, such as RBMX/hnRNP G (Adamson et al., 2012), p54(nrb) (also known as NONO; Krietsch et al., 2012), FUS (Mastrocola et al., 2013) or hnRNP U (Britton et al., 2014), associate with DNA lesions through the interaction with PAR chains. Interestingly, the interaction between PAR chains and some of these SFs is mediated by their RNA binding motifs, such as the arginine/glycine rich motif 2 (RGG2) of FUS (Mastrocola et al., 2013) or the RNA-recognition motif 1 (RRM1) of p54(nrb), whose binding to PAR polymers competes with their target RNA molecules (Krietsch et al., 2012). It is thus tempting to hypothesize that the increased levels of PAR polymers elicited by DNA lesions may redirect SF activity from mRNA processing toward DNA repair.

Interaction with PAR chains may not be the only factor regulating SFs recruitment on chromatin after DNA damage. For instance, recruitment of RBMX/hnRNP G to DNA lesions is unaffected by PARP inhibition (Adamson et al., 2012). Although no direct evidence is still available, one possibility is that additional histone marks can play a role in bridging RBPs to the DDR. Indeed, it is now well established that multiple histone modifications affect recruitment of SFs to target genomic locations (Iannone and Valcárcel, 2013). Thus, it is possible that the orchestrated epigenetic rearrangement elicited by the DDR (Papamichos-Chronakis and Peterson, 2013) may also affect recruitment of selected SFs at sites of damage. In line with this suggestion, recent reports have shown that trimethylated at Lys36 histone H3 (H3K36me3), which acts as docking site for the recruitment of SFs like hnRNP I/PTB and SRSF1 (Luco et al., 2010; Pradeepa et al., 2012), is an important epigenetic mark regulating both DNA mismatch and DSB repair through HR (Li et al., 2013; Pfister et al., 2014). It is therefore attracting to speculate that a complex interplay between DDR signaling proteins, epigenetic histone modifications surrounding the lesions and RBPs recruited to these sites ensures robust and efficient repair of the damage. Once recruited on the chromatin, RBPs can participate to repair of the DNA damage through splicing-unrelated functions that can be mainly classified into either sensor activity of the lesion or regulation of the activity of the repair machinery.

#### Splicing Factors Acting as Sensors of DNA Damage

A well-described example of DNA damage molecular sensor is the SF PRP19, a major component of the larger heteromeric protein complex called NTC/nineteen complex or PRP19 complex, which enhances the molecular rearrangements necessary for proper assembly and activity of the spliceosome (Chanarat and Sträßer, 2013). PRP19 has been recently proposed to sense single-strand DNA breaks (SSBs) through its interaction with proteins of the replication protein A (RPA) complex, which coat and stabilize single-strand DNA molecule (Maréchal et al., 2014; Wan and Huang, 2014). PRP9, a E3-ubiquitin ligase, ubiquitinases RPA creating docking sites for the recruitment of ATR-interacting protein (ATRIP), which in turn enhances the activation of its partner kinase Ataxia Telangiectasia and Rad3-related protein (ATR; Maréchal et al., 2014; Wan and Huang, 2014). Another interesting example of RBP involved in signaling DNA damage is FUS, which was observed to be recruited at DSBs in a PARP-dependent manner (Mastrocola et al., 2013). FUS-silenced cells were less proficient in repairing these lesions, as a consequence of an impairment of the early steps of the induction of the DDR (Mastrocola et al., 2013; Wang et al., 2013). Accordingly, recruitment of FUS at DSBs is an early event following DNA damage, which precedes and enhances histone H2AX phosphorylation and the subsequent recruitment of DDR signaling proteins (Wang et al., 2013). FUS ability to enhance the DDR also relies on its interaction with histone deacetylase 1 (HDAC1), whose recruitment and stable retention at the lesion site is necessary for proper DNA repair (Wang et al., 2013). Intriguingly, several of the mutations in the *FUS* gene identified in familial cases of amyotrophic lateral sclerosis (F-ALS) are located in the regions implicated in the interaction with HDAC1. Higher levels of γH2AX have been observed in the motor cortex of F-ALS patients carrying these mutations (Wang et al., 2013). Moreover, high levels of γH2AX have also been recently observed in both cortex and spinal cord of transgenic mice expressing one the most common F-ALS mutation, FUS-R521C (Qiu et al., 2014). The inability of this FUS mutant to interact with HDAC1 (Qiu et al., 2014) strongly suggests that the interaction between these two proteins is necessary for the proper induction of the DDR also in the brain of animal models. Together with the severe neurological and motor coordination defects observed in FUS-R521C transgenic mice, these observations may suggest that impairment of a proper DDR caused by mutations in FUS is involved in F-ALS pathogenesis.

Collectively, the studies summarized above highlight the important role played by RBPs as sensors of the damage and regulators of DDR activation, suggesting that they may be implicated in important pathologies correlated with DDR deficiencies.

#### Splicing Factors Regulating DNA Repair Processes

In addition to acting as sensors or enhancers of the DDR signaling pathway, several SFs have also been demonstrated to directly regulate the activity of protein complexes and enzymes mediating the DNA repair (Figure 1C). For example, the interaction between PSF [also known as splicing factor proline and glutamate-rich (SFPQ)] and RAD51 was shown to enhance the strand-exchange activity of this recombinase during HR-guided repair (Morozumi et al., 2009). Consistently, PSF silencing was reported to enhance cellular sensitivity to DNA crosslinking and alkylating agents and to reduce HR-mediated repair of DSBs (Rajesh et al., 2011). Similarly, the interaction between the SF hnRNP U and the DNA glycosylase NEIL1 stimulates the activity of this enzyme, which is responsible for the recognition and removal of oxidized DNA bases. Reducing the expression levels of both proteins synergically increased cellular sensitivity to oxidant agents, indicating the functional cooperation of this RBP with NEIL1 (Hegde et al., 2012). The interaction between SFs and DNA repair complexes may also inhibit their activity. For instance, the interaction between YB-1 and proliferating cell nuclear antigen (PCNA) impairs the formation of the MutSα-PCNA dimer, a component of the MSH6-mismatch-repair complex, and its recruitment at the mismatches, thus inhibiting DNA repair (Chang et al., 2014).

Beside these regulatory activities relying on the interaction with DRPs, SFs may also directly participate to DNA repair processes, as demonstrated for RBMX/hnRNP G and the heterodimer composed by PSF and p54(nrb). RBMX/hnRNP G binds DSBs to protect them from further degradation and stimulates the fidelity of the non-homologous end joining (NHEJ) system repair (Shin et al., 2007). The heterodimer PSF-p54(nrb) was instead shown to stimulate NHEJ *in vitro* and to directly bind DNA and to cooperate with Ku protein in the formation of a pre-ligation complex (Bladen et al., 2005).

**FIGURE 2:**
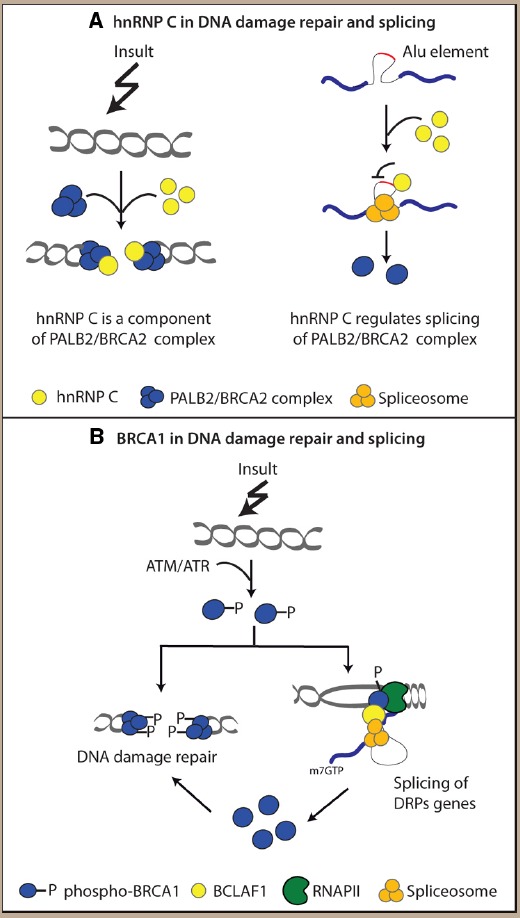
**Examples of interplay between splicing factors (SFs) and DNA repair proteins (DRPs). (A)** hnRNP C is recruited with the PALB2/BRCA2 complex at the DNA damage sites and it regulates proper splicing of its components. **(B)** Phosphorylated BRCA1 (pBRCA1) is recruited at the DNA lesions and it interacts with spliceosomal proteins favoring the correct splicing of DRPs encoding genes which sustain DNA damage response (DDR).

An intriguing possibility is that eukaryotic cells may exploit both the DNA repair and the splicing activity of a RBP to properly respond to the DNA damage. This hypothesis is supported by a study showing that hnRNP C is recruited at the DNA damage sites as part of the PALB2/BRCA2 nucleoprotein complex (Anantha et al., 2013). At the same time, however, hnRNP C also regulates correct expression of components of this complex by avoiding the exonization of Alu elements within their intronic sequence (Anantha et al., 2013; see Figure 2A). Thus, the dual activity of RBPs might help the cell to finely coordinate gene expression and DNA repair in order to overcome the DNA damage and to survive.

## DNA Repair Proteins Acting as Splicing Regulators

The interplay between splicing regulation and DDR might also take advantage of the multifunctional properties of some proteins involved in the repair of the lesion. Indeed, DNA repair proteins (DRPs) have been recently shown to play also a direct role in the regulation of pre-mRNA splicing.

In this regard, a major role is played by the protein kinases regulating the signal transduction pathway that trigger the DDR, such ATR, Ataxia Telangiectasia mutated (ATM) and by their downstream effectors, such as CHK1 or CHK2 (Figure 1D). As mentioned above, SFs and RBPs represent a major target of phosphorylation in response to genotoxic stress (Beli et al., 2012), often as direct targets of ATM/ATR (Matsuoka et al., 2007; Stokes et al., 2007). The phosphorylation status affects the expression, localization and activity of most SFs (Naro and Sette, 2013). Thus, it is likely that the widespread reprogramming of splicing profiles occurring during genotoxic stress (Sprung et al., 2011) may be partially due to the activity of the DDR-activated kinases. A well-described example of DDR kinase regulating pre-mRNA splicing is the serine-threonine kinase CHK2, a downstream effector of ATM (Ciccia and Elledge, 2010). Following exposure to genotoxic stresses, activation of CHK2 regulates AS of the *Taf1* gene in *Drosophila* and of the *BCL-X* gene in human cancer cells (Katzenberger et al., 2006; Shkreta et al., 2011). More recently CHK2 was shown to phosphorylate the splicing regulator HuR in response to oxidative stress (Akaike et al., 2014). This phosphorylation enhances HuR affinity for a region within exon 2 of the *TRA2*β pre-mRNA, thereby favoring its inclusion in the mature transcript. This splicing event leads to production of a shorter TRA2β isoform having multiple premature stop codons, which leads to a significant reduction of TRA2β protein levels following oxidative stress (Akaike et al., 2014). Notably, CHK2 was shown to regulate the splicing process also in absence of DNA damage, as it interacts with and mediates the constitutive phosphorylation of the splicing regulator CDK11p110, an important event for the proper efficiency of the splicing process (Choi et al., 2014).

DDR kinases mediated phosphorylation of SFs does not represent the only level of interplay between the DDR and the splicing process. Recent evidence suggests a direct role of DRPs in splicing regulation (Figure 1D), thus making this scenario far more complex than previously thought. For example, the polymerase delta interacting protein 38 (PDIP38), a protein involved in trans-lesion DNA synthesis following damage (Tissier et al., 2010), relocalizes in nuclear speckles following UV treatment and is important for the UV-induced AS of the *MDM2* gene (Wong et al., 2013). Another striking example of DRP that directly regulates the splicing process is BRCA1, a key regulator of genome integrity, playing an important role in different DNA repair pathways (Roy et al., 2011). A mass-spectrometry analysis aimed at identifying proteins that interact with BRCA1 following its phosphorylation by ATM led to the identification of BCL2 associated transcription factor 1 (BCLAF1; Savage et al., 2014), a protein that interacts with the core splicing machinery (Merz et al., 2007) and is involved in splicing regulation (Bracken et al., 2008). Interestingly, phosphorylated BRCA1 (pBRCA1) was shown to interact with several SFs, such as PRP8, U2AF65, U2AF35 and SF3B1, following DNA damage and in BCLAF1-dependent manner (Savage et al., 2014). Moreover pBRCA1 localization was not restricted to DNA damage sites, but the protein was also recruited at promoters of genes involved in DNA replication, recombination, and repair (Savage et al., 2014). In this way, pBRCA1 recruits BCLAF1 and spliceosomal components to these genes, thus ensuring their correct splicing and expression under stress conditions (Figure 2B). This mechanism would allow sustained expression of these proteins, which display high turnover rate, and promote DNA repair and survival of the cell (Savage et al., 2014).

These observations together with other findings highlighting the involvement of DRPs also in other aspects of mRNA metabolism, such as pre-mRNA 3′ processing (Kleiman et al., 2005) or mRNA stability control (Reinhardt et al., 2010), strongly suggest that eukaryotic cells may exploit these proteins also as non-canonical post-transcriptional regulators. Thus, DRPs are likely to be directly involved in the broad rearrangement of gene expression occurring during DDR, which ensures the proper expression of genes involved in DNA repair, cell cycle control and apoptosis regulation.

## Concluding Remarks

Preventing and resolving damage of the DNA is a priority for the cell, as integrity of the genome is a determining factor for cell survival and to avoid pathological situations. Therefore, as illustrated in the examples herein reviewed, cells employ the activity of many proteins, involved in different cellular processes, to guarantee genome stability. Beyond their known role as post-transcriptional regulators of gene-expression in response to DNA damage (Lenzken et al., 2013), splicing-unrelated functions of RBPs are also utilized for the prevention of transcription-coupled DNA damage and to ensure correct cellular euploidy. Integration of the splicing and genome gatekeeper activities of RBPs is certainly favored by their strategic interaction with both chromatin and nascent transcripts, which conceivably allows a quick shift and efficient coordination among these different functions according to the momentary cellular needs. Likewise, DRPs display an unexpected function in the post-transcriptional regulation of gene expression, which is also aimed at sustaining the DDR. It is reasonable to believe that this dual function of DRPs offers the advantage to promptly activate the required gene expression response required to overcome the DNA insults soon after detection. Overall these unpredicted findings have recently unveiled a tight interplay between splicing regulation and canonical DNA safeguard mechanisms, whose regulators may play dual functions in both processes. Full elucidation of these dual functions of SFs and DRPs is thus necessary to shed light on the complexity and the extent of this interplay.

Aberrant expression and activity of SFs have been clearly demonstrated as contributing factors in human cancers. Some efforts have been made during the last years to develop efficient therapeutic tools targeting their splicing activity or the activity of the aberrant splice variants they generate (Singh and Cooper, 2012; Havens et al., 2013). Nevertheless, the observations reported herein strongly suggest that the splicing unrelated functions of RBPs must also be taken into account to counteract their oncogenic activity. Functions of SFs as genome gatekeeper represent, therefore, an intriguing target for the development of new therapeutic approaches, which could be used in combined therapy to increase the radio- and or chemosensitivity of cancer cells, as it is already done for the inhibitors of canonical DRPs (Furgason and Bahassi, 2013).

### Conflict of Interest Statement

The authors declare that the research was conducted in the absence of any commercial or financial relationships that could be construed as a potential conflict of interest.
